# Supplementary material for: The marginal cells of the Caenorhabditis elegans pharynx scavenge cholesterol and other hydrophobic small molecules
Source: Nat Commun. 2019 Sep 2;10:3938. doi: 10.1038/s41467-019-11908-0 (PMC6718421; doi:10.1038/s41467-019-11908-0)
Supplement: Supplementary file 1 — Supplementary Information [file 41467_2019_11908_MOESM1_ESM.pdf]

Supplementary Information

The Marginal Cells of the *Caenorhabditis elegans* Pharynx Scavenge Cholesterol and Other Hydrophobic Small Molecules

Kamal *et al.*

Name[a]	Chembridge ID	logSw	RB	tPSA	Hacc	Hdon	MW	% object	s.e.m.
**Molecules with Properties coincident with the Crystalizing Class**									
3-(3-methoxyphenyl)-5-methyl-7H-furo[3,2-g]chromen-7-one	9006392	-5.09	1	52.58	4	0	306.312	0	0
N,N-dimethyl-4-[(3-methyl-4-nitro-1,1-dioxido-2(5H)-thienylidene)methyl]aniline	5319944	-5.14	1	80.52	4	0	308.353	0	0
2-(4-fluorophenyl)-4-(2-nitrobenzylidene)-1,3-oxazol-5(4H)-one	5924202	-5.20	2	81.80	5	0	312.252	0	0
5-(5-bromo-2-furyl)-3-(3-methylphenyl)-1,2,4-oxadiazole	7342285	-5.22	2	52.06	4	0	305.127	38.33[b]	10.18
3-(3-ethoxy-4-methoxyphenyl)-5-(3-methylphenyl)-1,2,4-oxadiazole	7983170	-5.28	2	57.38	5	0	310.347	0	0
4-methyl-2-(4-methyl-1-piperidinyl)-5-nitro-6-(1-pyrrolidinyl)pyrimidine	7399278	-5.37	2	75.40	4	0	305.375	39.54[b]	10.47
4-(1-piperidinylcarbonyl)-9H-fluoren-9-one oxime	6664420	-5.38	1	52.90	3	0	306.358	0	0
4-chloro-1-(2,4-dichlorobenzyl)-3-nitro-1H-pyrazole	7903521	-5.41	2	60.96	3	0	306.533	0	0
3-(2-thienylcarbonyl)naphtho[1,2-b]furan-4,5-dione	5169033	-5.66	2	64.35	4	0	308.308	0	0
11-(2-methyl-1H-imidazol-1-yl)-2,3-dihydro-1H-cyclopenta[4,5]pyrido[1,2-a]benzimidazole-4-carbonitrile	5884813	-5.72	1	58.91	5	0	313.356	0	0
**Molecules with Properties coincident with the Non-Object Forming Class**									
3-(4-bromo-2,6-dimethylphenoxy)propyl]methylamine hydrochloride	7707521	-3.62	4	21.26	2	1	308.642	0	0
N-[4-(4-ethyl-1-piperazinyl)phenyl]benzamide	9013406	-3.65	3	35.58	2	1	309.405	0	0
4-(dimethylamino)benzyl][2-(4-fluorophenyl)ethyl]amine hydrochloride	9009682	-3.69	5	15.27	1	1	308.821	0	0
N-(3-ethoxybenzyl)-2-(4-fluorophenyl)ethanamine hydrochloride	9022417	-3.74	5	21.26	2	1	309.806	0	0
N-cyclopropyl-4-(3,4-dihydro-2(1H)-isoquinolinylmethyl)benzamide	6410690	-3.75	4	32.34	2	1	306.401	0	0
1-methyl-N-{3-[(4-methylphenyl)thio]propyl}-3-piperidinecarboxamide	9306278	-3.89	6	32.34	2	1	306.466	0	0
1-benzyl-N-[2-(methylthio)phenyl]-4-piperidinamine	5477104	-3.92	4	15.27	1	1	312.472	0	0
4-(4-methylphenyl)-5-(4-thiomorpholinylmethyl)-2,4-dihydro-3H-1,2,4-triazole-3-thione	5592003	-3.95	3	36.85	2	1	306.45	0	0
N-phenyl-2-(4-phenyl-1-piperazinyl)propanamide	9102668	-3.99	4	35.58	2	1	309.405	0	0
N-(sec-butyl)-1-(3-chlorobenzyl)-4-piperidinecarboxamide	6581023	-4.00	5	32.34	2	1	308.846	0	0

Supplementary Table 1.

[a]The crystal-forming ability of 10 new molecules that have physicochemical properties that are consistent with crystal formation and 10 new molecules that have physicochemical properties that are consistent with no-object formation. See Crystal and Sphere Analyses Methods for details. ID, catalog number; SW, solubility in water coefficient; RB, rotatable bonds; tPSA, topological polar surface area; Hacc, Hdon, hydrogen bond donors and acceptors, respectively; MW, molecular weight; s.e.m., standard error of the mean.

[b]That 20% of the molecules with crystal-like physicochemical properties form crystals is a 147-fold enrichment over what is expected by random chance. The enrichment is calculated relative to the crystal-forming compounds discovered in our screen of 67012 small molecules (Burns *et al.,* 2019). In our current manuscript, we provided multiple lines of evidence that support that crystal-forming compounds kill animals. From our screen of 67012 small molecules, we found that 275 molecules kill *C. elegans*. Hence, at most, there are 275 molecules in the set of 67012 molecules that crystalize. However, we can further refine our estimate of the number of crystalizing compounds in the set of 67012 screened molecules as follows: 38 of the 238 molecules that we surveyed form crystals (Figure 1a). These 238 molecules contained 115 of the 275 lethal molecules that we found in our screen of 67012 molecules (see Burns *et al.*, 2015). Given that our data indicate that crystal-forming compounds kill worms, we can extrapolate that 90.9 of the 275 lethals form crystals ((38/115)x275). Given that non-lethal molecules do not form crystals, we can assume that at most, 90.9 of the 67012 molecules screened form crystals. Hence our hit rate of 2 out of 10 molecules forming crystals is a 147 fold enrichment of crystal forming compounds over our original screen ((2/10):(90.9/67012)). Source data are provided in the Source Data file.

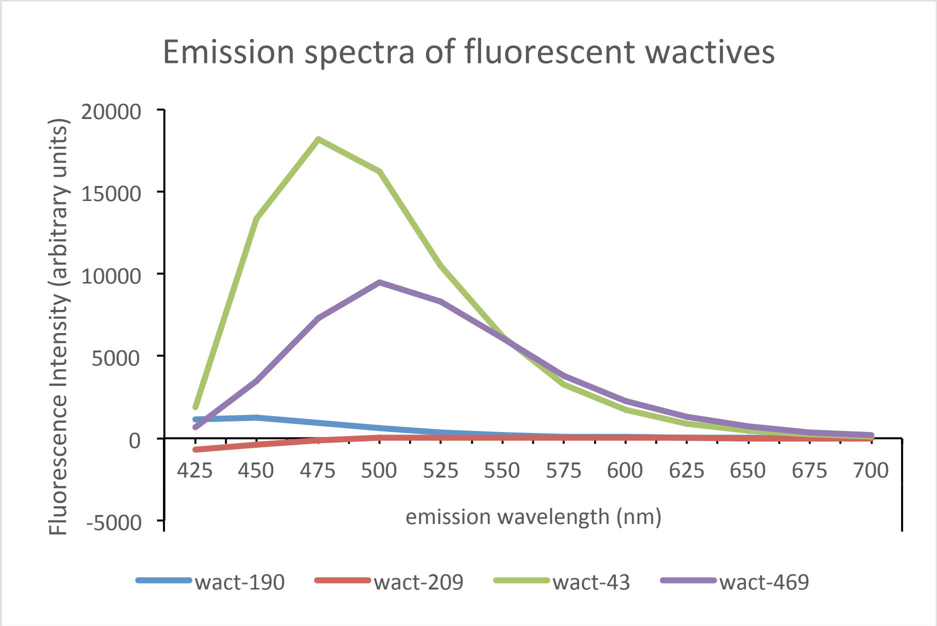

Supplementary Figure 1. Fluorescent Properties of wact-190, wact-469, wact-209, and wact-43. Wactive compounds were analyzed at a final concentration of 250 mM in 50 mL of double-distilled water in a 96-well flat-bottom plate (Corning). A fluorescence intensity emission scan was performed by exciting the compounds at 390 nm. The resulting emission spectrum was plotted for each compound.

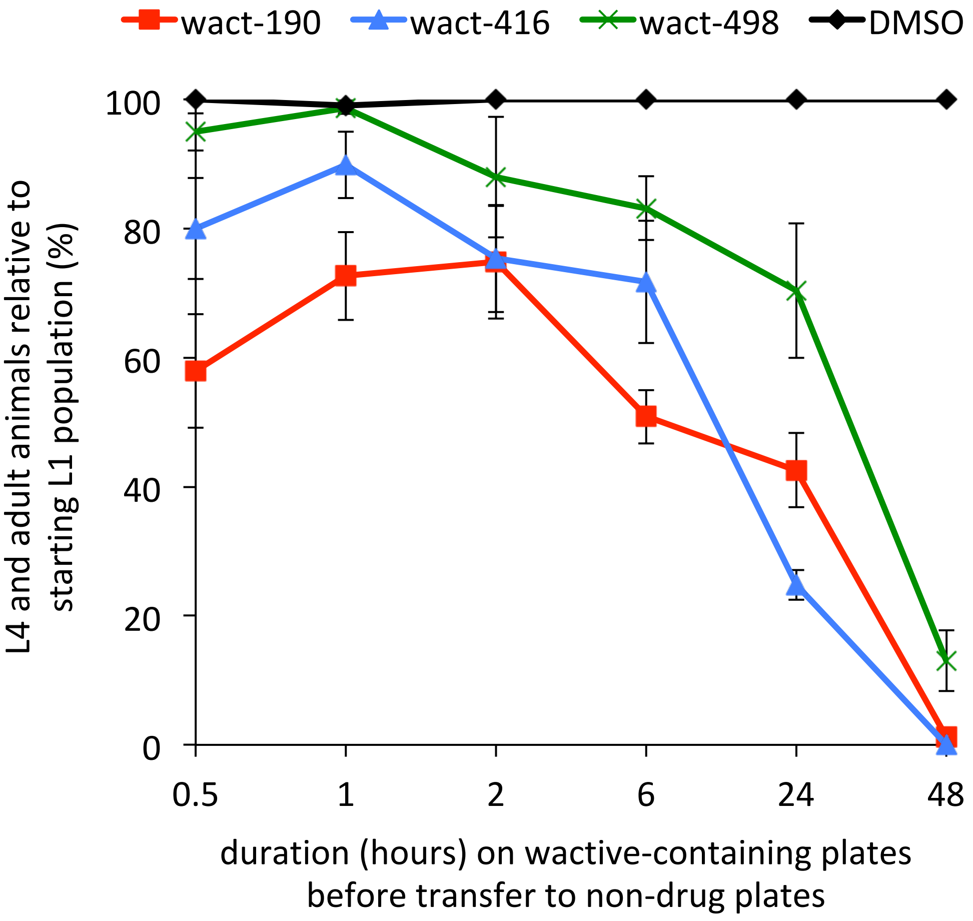

Supplementary Figure 2. An Analysis of the Time Needed for Crystal-Forming Wactives to Kill *C. elegans*. Synchronized L1s were incubated in 30 µM each wactive (or 1% DMSO control) using 50 L1s/well (4 technical replicates, 2 biological replicates) for the indicated duration of time. Thereafter, the worms were transferred to non-drug plates (t=0) and the number of L1s were counted 24 hours later (t=24). The number of animals that reach L4 or adulthood was counted 48 hours later (t=72) and expressed as a fraction of total number of L1s counted at t=24. See methods for additional details. Standard error of the mean is shown. Source data are provided in the Source Data file.

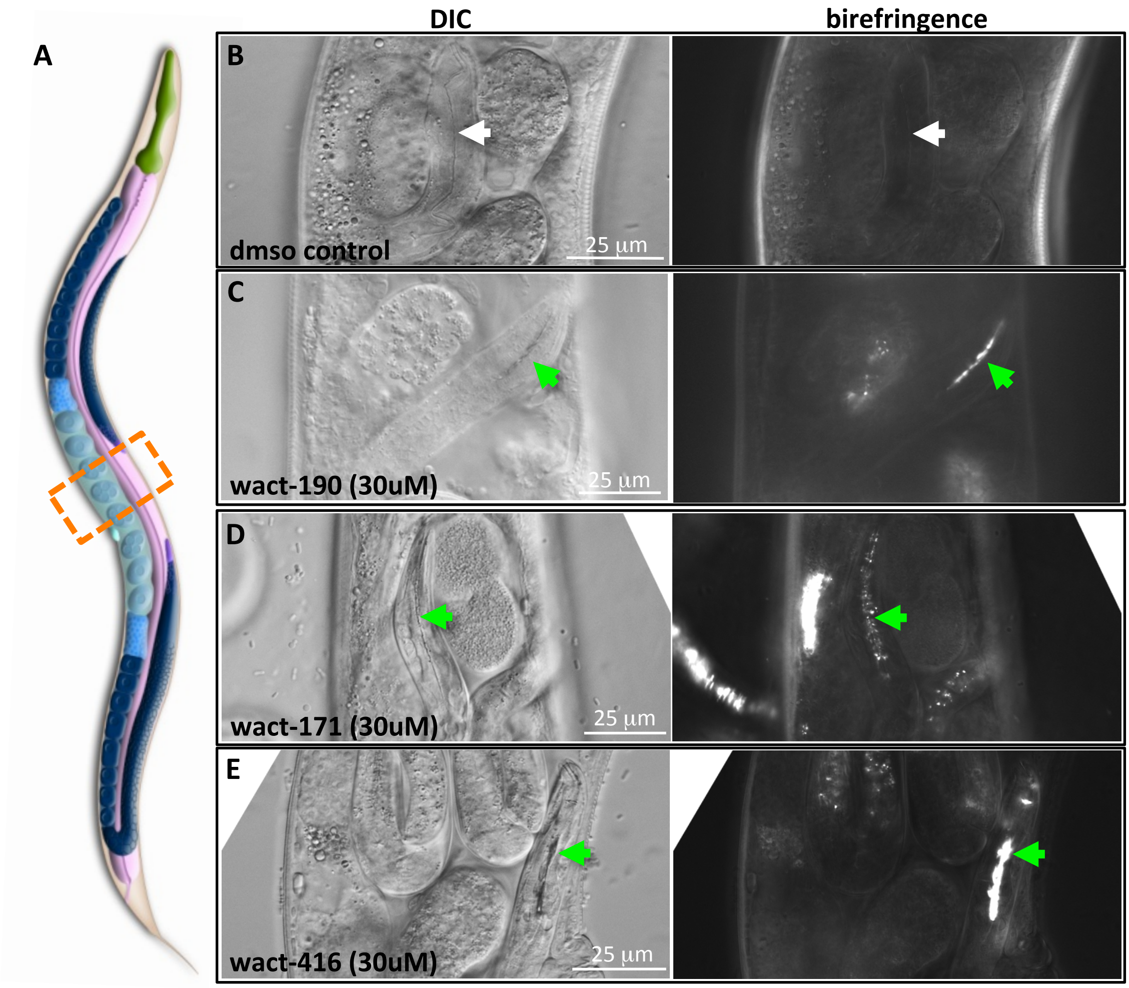

Supplementary Figure 3. Animals that Hatch Internally can Accumulate Crystals. A. A schematic of *C. elegans* (with permission from WormAtlas). The orange box highlights the approximate area shown in figures B-E. **B-E.** Differential interference contrast (DIC) are shown on the left column and the corresponding birefringent images are shown on the right. All animals shown were grown in liquid as synchronized L4s, incubated in either vehicle control (1% DMSO), or the indicated wactives, for 48 hours. The anterior pharynx of the internal hatchlings is indicated with a white arrow (no obvious objects) or a red arrow (where crystals are evident).

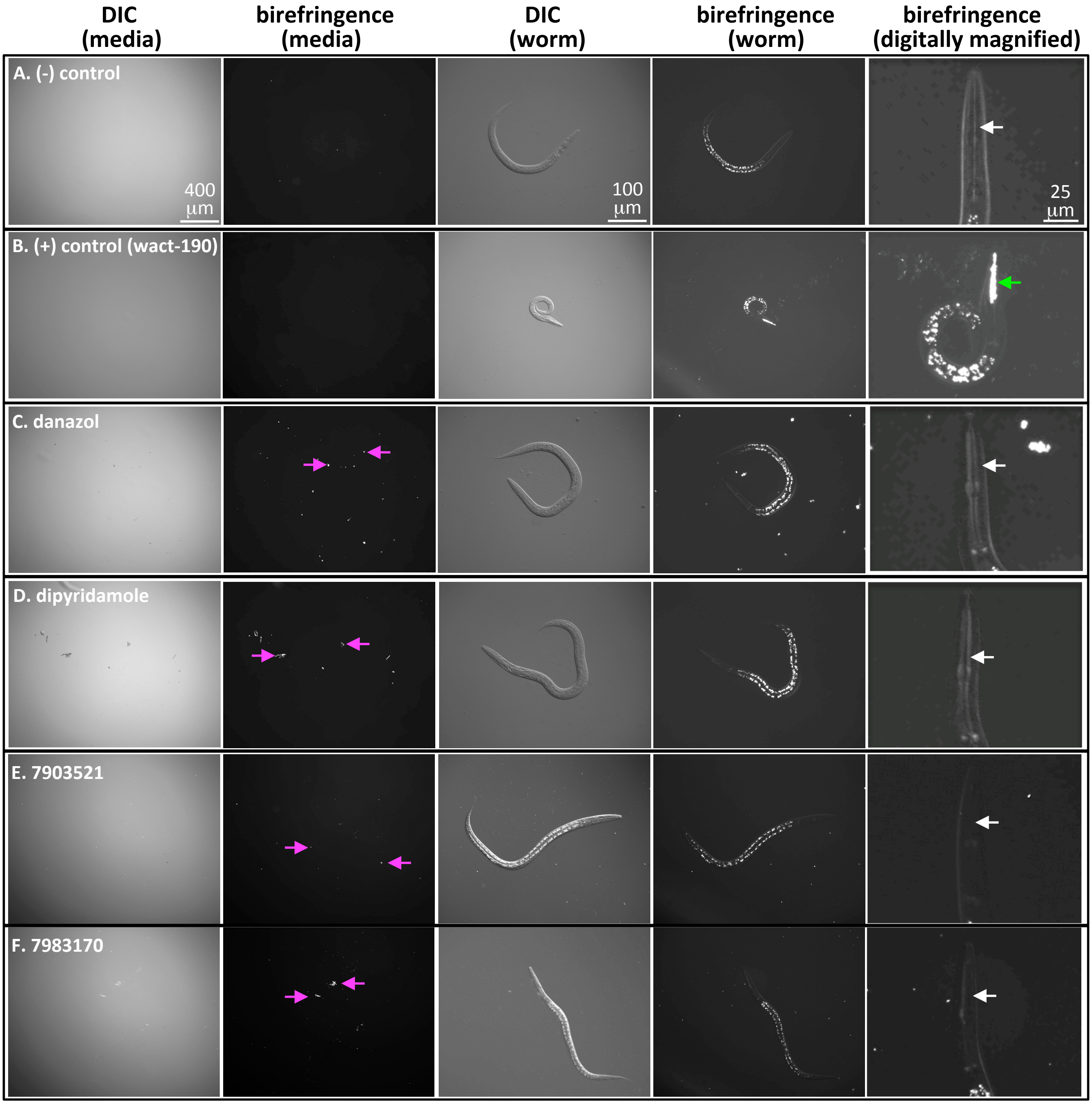

Supplementary Figure 4. See next page for legend.

Supplementary Figure 4. Crystal Precipitate in the Media is Not Sufficient for Crystal Formation within the Anterior Pharynx. Shown are four examples of the 17 molecules that we investigated for crystal formation in the anterior pharynx (or any other tissue). Each row highlights a single compound or control. '**A**', the negative control (the solvent DMSO); '**B**', wact-190 is a (+) control for crystal formation in the pharynx, but lacks any obvious precipitated in the media. **C-F**, examples of worms incubated in the indicated molecules, none of which lead to object formation in the anterior pharynx (or anywhere else). 'E-F' are Chembridge Inc molecules (the catalog number is indicated). In addition to these four molecules, the 13 other molecules that we examined and form precipitate in the media but not in the pharynx include, danthron, cortisone acetate, dienestrol, estradiol cypionate, fluocinonide, iodoquinol, medroxyprogesterone acetate, and the Chembridge molecules 5169033, 5319944, 5884813, 5924202, 6664420, 9006392. The first two columns show a low magnification image of only the media, the crystal precipitate in which can be clearly seen by birefringence (pink arrows highlight some precipitate). Crystal precipitate sometimes carries over when mounting worms (third and fourth columns). The fifth column is a digitally magnified image. The green arrow shows crystalized molecule in the antieror pharynx. The white arrows indicate the anterior pharynx without crystal formation. Assays were carried out in the same way as our initial survey of 238 molecules (see methods). Source data are provided in the Source Data file.

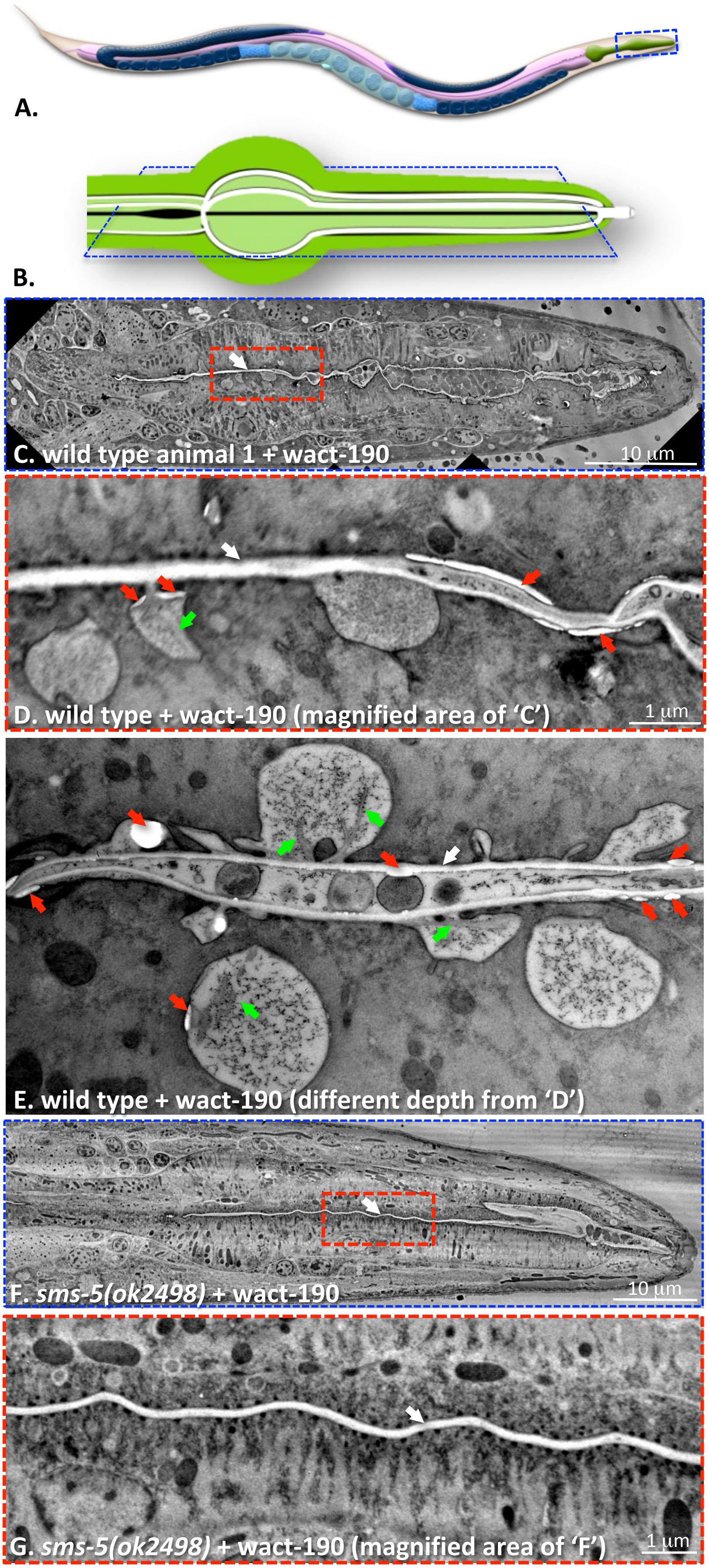

Supplementary Figure 5. Transverse Transmission Electron Microscopy (TEM) Sections of Animals Co-Incubated with wact-190. A & B. Schematics illustrating the area of the worm and the sectioning shown in **C** and **F**. **C-E.** A wild type L1 animal incubated in 60 mM of wact-190 for 24 hours. White arrows indicate apical membrane of the marginal cells, green arrows indicate crystal-like objects, and red arrows indicate unusual space that is consistent with either wact-190 accumulation or tissue separation due to wact-190 treatment. **D** is an enlarged area boxed in 'C'; **E** is another focal plane of the pharynx of the same animal. Given that we fail to see wact-190-related spheres *in vivo*, we interpret the circular inclusions in the transverse T.E.M. images to be caused by crystal growth. Most or all crystals are observed to lie at cell-cell borders in the apical zone of the anterior pharynx in the TEM analyses. In this region, the pharyngeal cells always meet in non-homologous pairings, as muscle cell-marginal cell appositions. Thus all crystals lie close to both cell types, but are observed to invaginate into the marginal cell rather than the muscle cell. Internal features (actinomyosin bundles vs intermediate filament bundles) help to some regard, but marginal cells always lie at the apex of narrow channels lining the lumen, while muscle cells make up the middle of the luminal wall. So, this geometry conclusively indicates that the crystal-type objects exist in the marginal cells and not the muscle cells. **F-G.** An *sms-5(ok2498)* mutant L1 animal incubated in 60 mM of wact-190 for 24 hours and then examined by TEM. **G** is an enlarged area boxed in **F**.

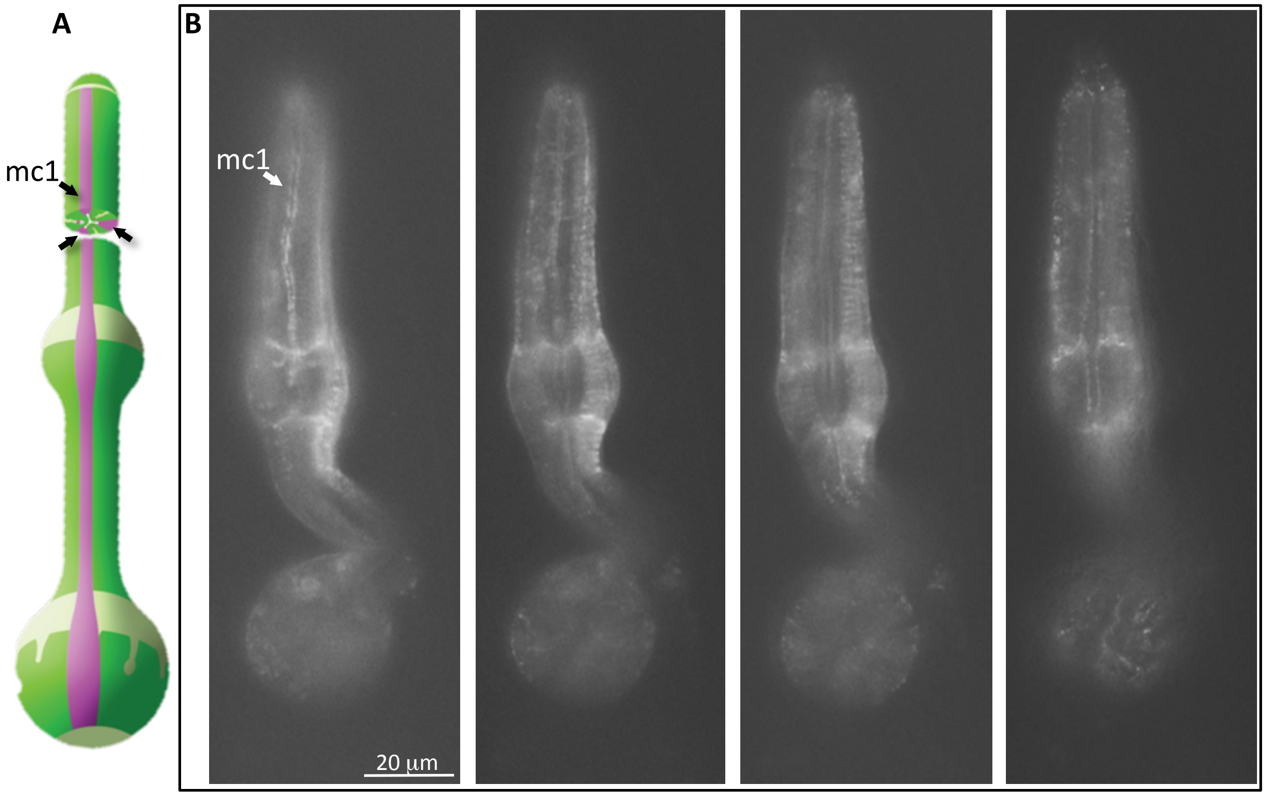

Supplementary Figure 6. The Expression Pattern of SMS-5 in the Pharynx. Animals harbouring an extrachromosomal array with the pPRHM1051 transgene (which has YFP inserted in frame at the C-terminus of the *sms-5* coding sequence in the context of the WRM0626dC03 fosmid) show fluorescent signal in only the pharynx and spermatheca (not shown). **A.** A schematic of the pharynx (courtesy of WormAtlas) indicating the marginal cells (pink) with black arrowheads. **B.** Four different focal planes of the pharynx of a non-mosaic animal expressing the SMS-5::YFP transgene. A marginal cell (mc1) is indicated with a white arrowhead.

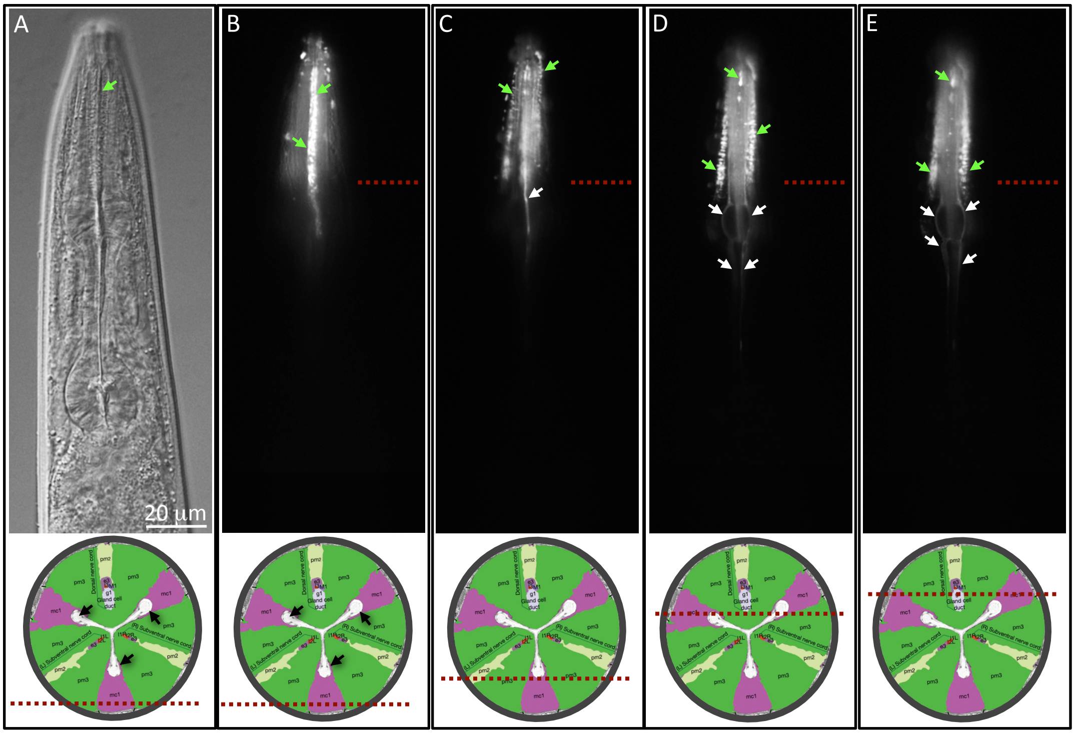

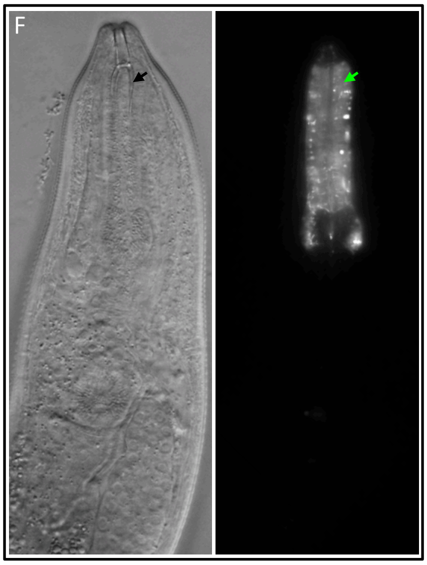

Supplementary Figure 7. The Characterization of a Marginal Cell-Specific Promoter. A. A DIC image at the focal plane of the image shown in B. **B-E.** Four focal planes of an animal harboring an extra-chromosomal array with pPRHM1053, a construct consisting of a genomic copy of *pgp-14* (fosmid WRM64dE02) with YFP coding sequence inserted in frame at the C-terminus of PGP-14. PGP-14::YFP is expressed exclusively in the marginal cells of the anterior pharynx (corpus). White arrows indicate the basolateral surface of the marginal cells and green arrows point to the apical (luminal) surface of the cells that line the channels. The rust-colored dotted line indicates the approximate cross-section represented in schematic beneath micrograph. Schematics are modified from Wormatlas. Marginal cells are purple; muscle is green, and the black arrows indicate the channels of the lumen. **F.** On the left, a DIC image of an animal expressing SMS-5::FLAG::mCherry from the anterior pharynx-marginal cell-specific *pgp-14* promoter. The black arrow indicates one of the channels. On the right, a fluorescent image of the red fluorescent channel of the same animal depicted on the left showing SMS-5::FLAG::mCherry expression (green arrow) that is restricted to the marginal cells of the anterior pharynx.

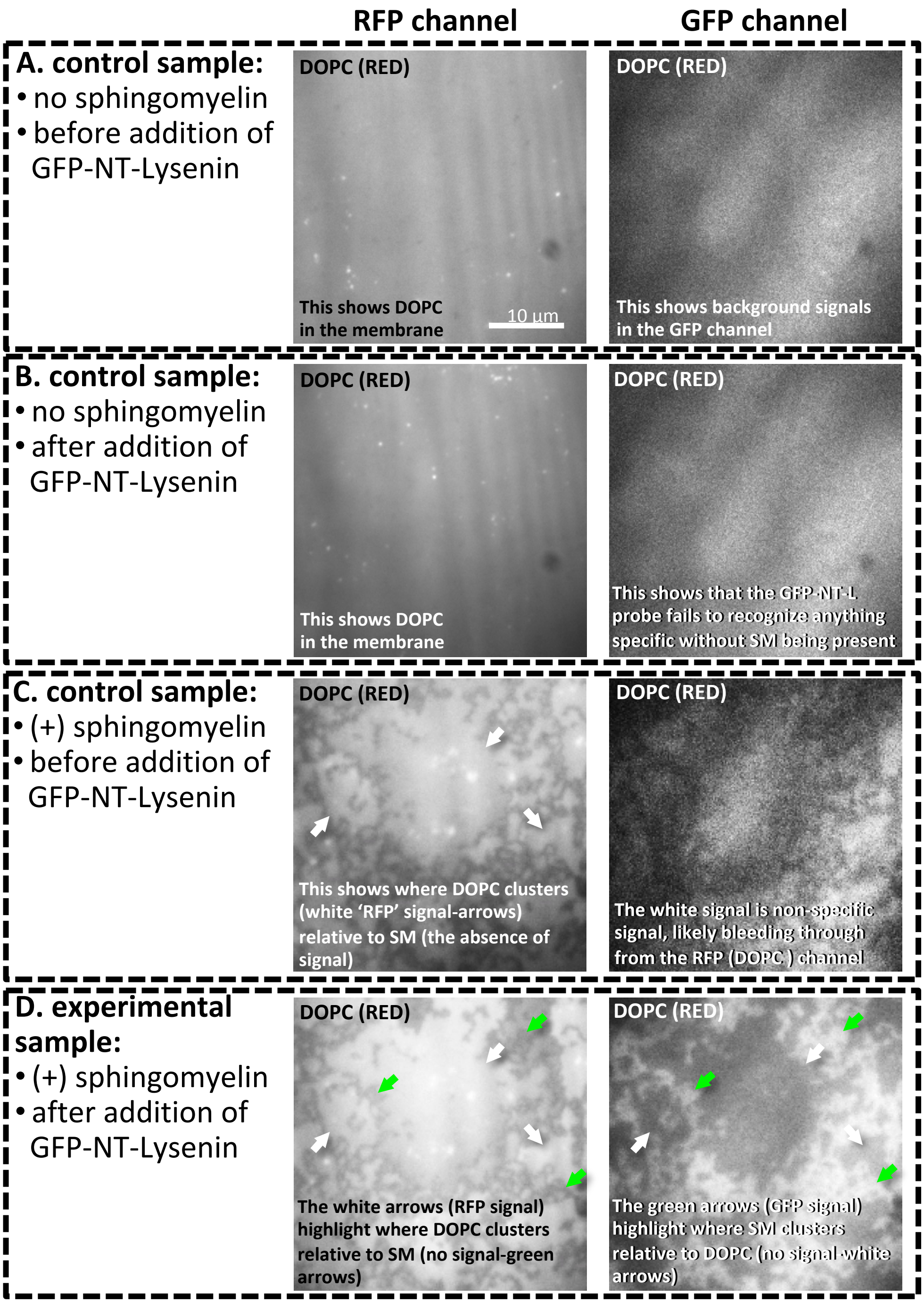

Supplementary Figure 8. A Control Experiment that Shows that the GFP-NT-Lysenin Probe Binds Clustered Sphingomyelin. A-B. The micrographs on the top row show a single lipid sample that is a suspended artificial 1,2-Dioleoyl-sn-glycero-3-phosphocholine (DOPC) lipid bilayer, either before (A) or after (B) the GFP-NT-Lysenin probe was added to the sample. All samples shown are stained with Dil C, which binds DOPC and fluoresces red. Fluorescent signal is seen in the RFP channel (left), while only background fluorescence is seen in the GFP channels (right). **C-D.** The micrographs on the bottom two rows show a single lipid sample of DOPC mixed with sphingomyelin (SM) and otherwise treated in the same way as described above in A & B. In the bottom row, homotypic clustering of the DOPC and SM lipids can be seen. DOPC clusters can be seen in the RFP channel (white arrows) (left) that are complementary to the GFP-NT-Lysenin fluorescent signal seen in the GFP channel (green arrows) after the GFP-NT-Lysenin probe (20µg/mL) has been added (15 minutes at room temperature) (D-right). The scale is the same for all panels.

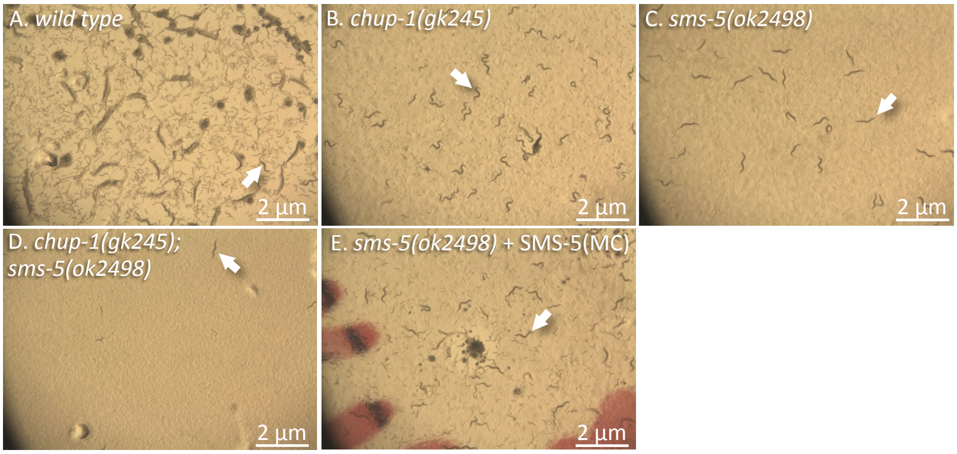

Supplementary Figure 9. Examples of Cultures of Strains Grown in Cholesterol-Limited Conditions. The micrographs show a sector of the surface of the petri dish on which the indicated strains are grown in cholesterol(xol)-limited conditions (containing 50 ng/mL xol), corresponding to the data reported in Figure 9a. Arrows indicate representative adult animals on each plate. SMS-5(MC) represents marginal-cell specific expression of SMS-5 from the *trIs104* integrated transgenic array. The rust-colored streaks in the background of 'E' are part of the labeling of the plate. See methods for additional details.

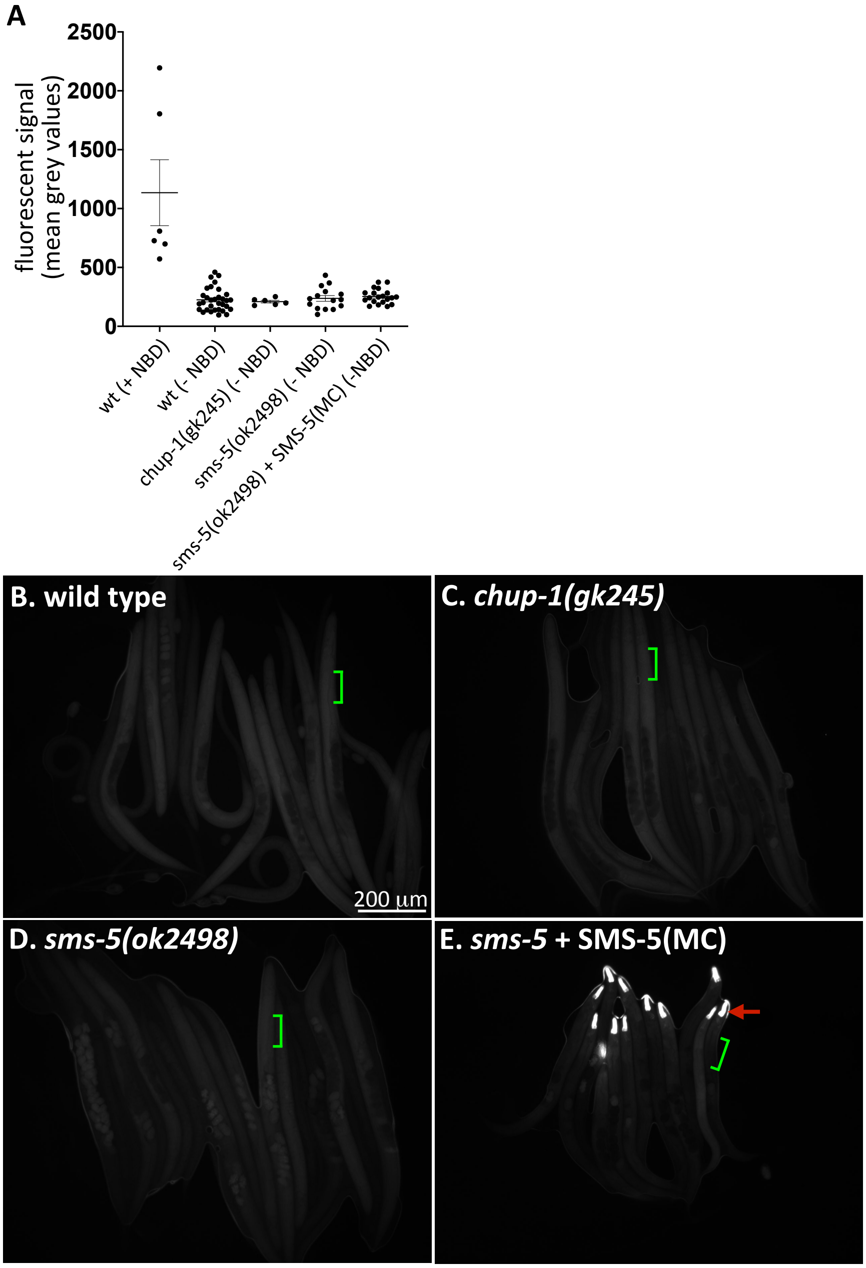

Supplementary Figure 10. Measurement of Auto-Fluorescent Signal in Strains Incubated without NBD-cholesterol. A. Auto-fluorescent signal of the indicated strains incubated without NBD-cholesterol (-NBD) compared to wild type incubated with NBD-cholesterol (+NBD) using the same filter sets as that used to analyze the NBD-cholesterol signal in Figure 9. There are no significant differences in background signal intensity in any of the (-NBD) mutant strains compared to the wild type incubated without NBD-cholesterol (p>0.25) using a Student's T-Test. Owing to its use as a control in several experiments, n=150 animals measured for wt(+)NBD; n=32 animals measured for wt (-)NBD; n=15 animals measured for *sms-5*(-)NBD; n=6 animals measured for *chup-1*(-)NBD; n=20 animals measured for *sms-5*+SMS-5*(MC)*(-)NBD. Standard error of the mean is shown. Source data are provided in the Source Data file. **B-E**. Images of multiple animals after the indicated strains were incubated without NBD-cholesterol for six days. The scale is the same for all images. Green lines exemplify the area used in each animal to calculate signal in 'A'. Red arrow indicates the expression of the YFP marker used to mark the presence of the rescuing transgene *trIs104*. In 'A' and 'E', SMS-5(MC) represents marginal-cell (MC) specific expression of SMS-5 from the *trIs104* integrated transgenic array.
